# Epidemiological Trends of Five Common Diarrhea-Associated Enteric Viruses Pre- and Post-Rotavirus Vaccine Introduction in Coastal Kenya

**DOI:** 10.3390/pathogens9080660

**Published:** 2020-08-15

**Authors:** Arnold W. Lambisia, Sylvia Onchaga, Nickson Murunga, Clement S. Lewa, Steven Ger Nyanjom, Charles N. Agoti

**Affiliations:** 1 Kenya Medical Research Institute (KEMRI)-Wellcome Trust Research Programme, Centre for Geographic Medicine Research-Coast, Kilifi 230-80108, Kenya; onchagasylvia@gmail.com (S.O.); nmurunga@kemri-wellcome.org (N.M.); clewa@kemri-wellcome.org (C.S.L.); cnyaigoti@kemri-wellcome.org (C.N.A.); 2 Department of Biochemistry, Jomo Kenyatta University of Agriculture and Technology, Juja 62000-00200, Kenya; snyanjom@jkuat.ac.ke; 3 School of Health and Human Sciences, Pwani University, Kilifi 195-80108, Kenya

**Keywords:** viral diarrhea, real-time PCR, rotavirus vaccination, Kenya

## Abstract

Using real-time RT-PCR, we screened stool samples from children aged <5 years presenting with diarrhea and admitted to Kilifi County Hospital, coastal Kenya, pre- (2003 and 2013) and post-rotavirus vaccine introduction (2016 and 2019) for five viruses, namely rotavirus group A (RVA), norovirus GII, adenovirus, astrovirus and sapovirus. Of the 984 samples analyzed, at least one virus was detected in 401 (40.8%) patients. Post rotavirus vaccine introduction, the prevalence of RVA decreased (23.3% vs. 13.8%, *p* < 0.001) while that of norovirus GII increased (6.6% vs. 10.9%, *p* = 0.023). The prevalence of adenovirus, astrovirus and sapovirus remained statistically unchanged between the two periods: 9.9% vs. 14.2%, 2.4% vs. 3.2 %, 4.6% vs. 2.6%, (*p* = 0.053, 0.585 and 0.133), respectively. The median age of diarrhea cases was higher post vaccine introduction (12.5 months, interquartile range (IQR): 7.9–21 vs. 11.2 months pre-introduction, IQR: 6.8–16.5, *p* < 0.001). In this setting, RVA and adenovirus cases peaked in the dry months while norovirus GII and sapovirus peaked in the rainy season. Astrovirus did not display clear seasonality. In conclusion, following rotavirus vaccine introduction, we found a significant reduction in the prevalence of RVA in coastal Kenya but an increase in norovirus GII prevalence in hospitalized children.

## 1. Introduction

In the year 2016 alone, approximately 300,000 children aged <5 years succumbed to diarrhea in sub-Saharan Africa [1]. Viral pathogens including rotavirus group A (RVA), adenovirus (type 40/41), astrovirus, norovirus (genogroup GI and GII) and sapovirus are among the top causative agents of severe diarrhea globally [2,3]. Understanding their epidemiological patterns such as prevalence, incidence, seasonality, clinical severity and infection age distribution in local settings is essential for designing and prioritizing interventions. Historically, RVA has been the single most important cause of severe childhood diarrhea, responsible for ~38% (95% CI: 4.8–73.4%) of hospital cases (<5 years) pre-vaccine introduction [4]. However, RVA prevalence has been rapidly declining since 2009 and was approximately 23% (95% CI: 0.7–57.7%) in 2016, in settings where the rotavirus vaccine was in use [4]. Due to the shared ecological niche and the apparent decline of all-cause gastroenteritis-associated hospital admissions, it has been hypothesized that rotavirus vaccination has likely impacted the epidemiology of the other enteric viruses [5]. However, there are contradicting reports on the specific impact of rotavirus vaccination on the prevalence of the individual enteric viruses—for example, norovirus [6,7]. This has not been adequately examined in African populations where diarrhea burden is highest. Kenya began rotavirus vaccination in July 2014 using the monovalent Rotarix^®^ (RV1), derived from G1P[8] strain, administered at 6 and 10 weeks of life. RV1 vaccine coverage in Kenya has increased over time since 2014 but is varied by age group, number of doses and geographic region in Kenya [8]. Within Kilifi County, coastal Kenya, coverage in 2017 in <1-year-olds was 73% (at least one dose) vs. 65% (complete two doses), while in <12–24 month-olds, it was 86% (at least one dose) vs. 84% (complete two doses) [9].

The KEMRI/Wellcome Trust Research Programme (KWTRP) has been running surveillance of RVA since 2002 in children admitted to the Kilifi County Hospital (KCH). The current study screened archived diarrheal samples from KCH, spanning both the pre- and post-rotavirus vaccine introduction periods in Kenya for RVA, astrovirus, adenovirus (all serotypes), sapovirus and norovirus (only GII) using real-time reverse-transcription polymerase chain reaction (RT-PCR) approach. We update on the prevalence of these viral diarrheal agents and their seasonal patterns pre and post introduction of the rotavirus vaccination program in Kenya.

## 2. Results

### 2.1. Study Population Characteristics

Out of 2156 children aged <5 years who presented with diarrhea at KCH during the four selected years (2003, 2013, 2016 and 2019), 1397 (64.8%) provided a stool sample; see Table 1. Overall, the demographic characteristics of the eligible children sampled, and eligible children not sampled, differed in age strata distribution (*p* = 0.001) and discharge outcome (*p* < 0.001); see Table 1. The main reasons for failure to sample eligible children were as follows: death (*n* = 21, 2.8%), discharge or transfer before sample collection (*n* = 296, 40.0%), consent refusal (*n* = 315, 41.5%) or other (*n* = 127, 16.7%). Among the sampled cases, 984 (70.4%) had a specimen available and tested by real-time RT-PCR for the five enteric viruses, and these were included in subsequent analysis. The median age of the sampled participants was significantly higher for the post-vaccine introduction period compared to pre-vaccine introduction period (*p* < 0.001); see Table 2.

### 2.2. Overall Virus Detection

**Figure 1:**
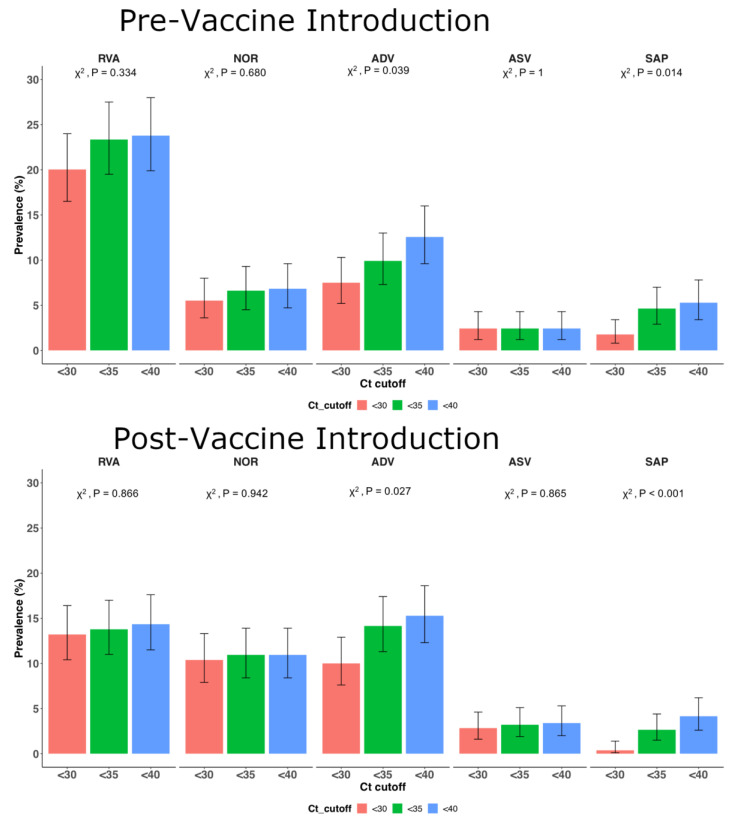
Detection frequency of RVA, adenovirus, norovirus GII, astrovirus and sapovirus at different cycle threshold (Ct) cutoffs for children under 5 years of age admitted to KCH Kenya with diarrhea symptoms. The error bars represent 95% confidence interval for the proportions. Proportions were compared using chi-square test. RVA stands for rotavirus group A, ADV stands for adenovirus, NOR stands for norovirus GII, ASV stands for astrovirus and SAP stands for sapovirus.

Of the 984 samples analyzed, at least one of the viruses was detected in 401 samples (40.8%) at the real-time RT-PCR cycle threshold (Ct) value of <35.0. The lower the Ct value, the higher the virus titer in the sample. The detection frequency differed significantly for adenovirus (*p* = 0.001) and sapovirus (*p* < 0.001) pre- and post-rotavirus vaccine introduction when the Ct cut-off value was gradually lowered (<30, <35, <40), unlike for RVA, astrovirus and norovirus GII; see Figure 1. All our subsequent analyses were undertaken at Ct value <35.0 Single infections were detected in 354 specimens (36.0%) and included RVA (*n* = 149, 42.1%), adenovirus (*n* = 91, 25.7%), norovirus GII (*n* = 75, 21.2%), sapovirus (*n* = 20, 5.7%) and astrovirus (*n* = 18, 5.1%).

### 2.3. Patterns Pre-Post Vaccine Introduction

**Figure 2:**
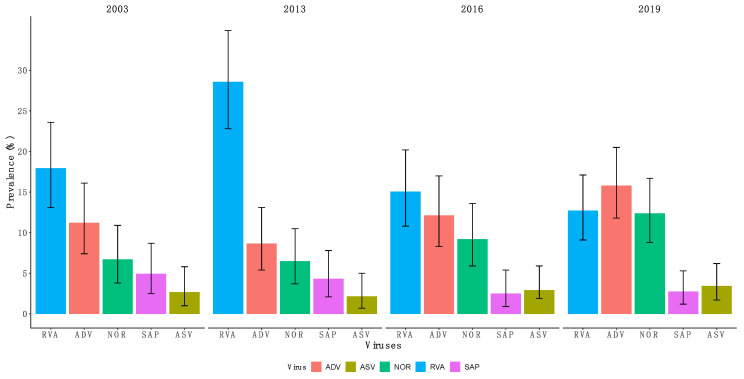
Prevalence of RVA, adenovirus, norovirus GII, astrovirus and sapovirus in 2003, 2013, 2016 and 2019 in children under 5 years of age admitted to KCH Kenya with diarrhea symptoms. The error bars represent 95% confidence interval for the proportions. Proportions were compared using chi-square test. Abbreviations used for viruses as in Figure 1.

RVA showed a significant decrease (23.3% vs. 13.8%, *p* < 0.001) in prevalence while norovirus GII showed a significant increase (6.6% vs. 10.9%, *p* = 0.02) post-vaccine introduction compared to pre-vaccine introduction; see Table 3. There were no significant changes in the prevalence of astrovirus (*p* = 0.585), adenovirus (*p* = 0.053) and sapovirus (*p* = 0.133) pre- and post-RVA vaccine introduction (chi-squared (χ^2^) test); see Table 3. Notably, norovirus GII had a gradual increase in prevalence across the four years, from 6.7% (95% CI: 3.8–10.9%) to 12.4% (95% CI: 8.8–16.7%); see Figure 2. RVA was the most commonly detected virus across all years, except in year 2019, in which adenovirus had the highest prevalence; see Figure 2.

**Figure 3:**
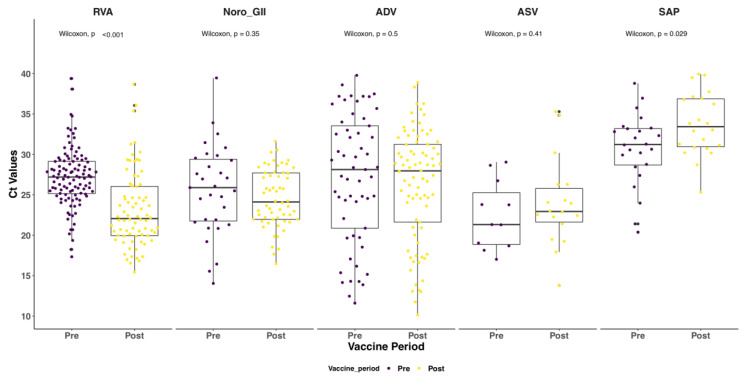
Distribution of Ct values among cases under 5 years of age admitted to KCH Kenya with diarrhea symptoms pre- and post-vaccine introduction. RVA stands for rotavirus group A, ADV stands for adenovirus, NOR GII stands for norovirus GII, ASV stands for astrovirus and SAP stands for sapovirus.

**Figure 4:**
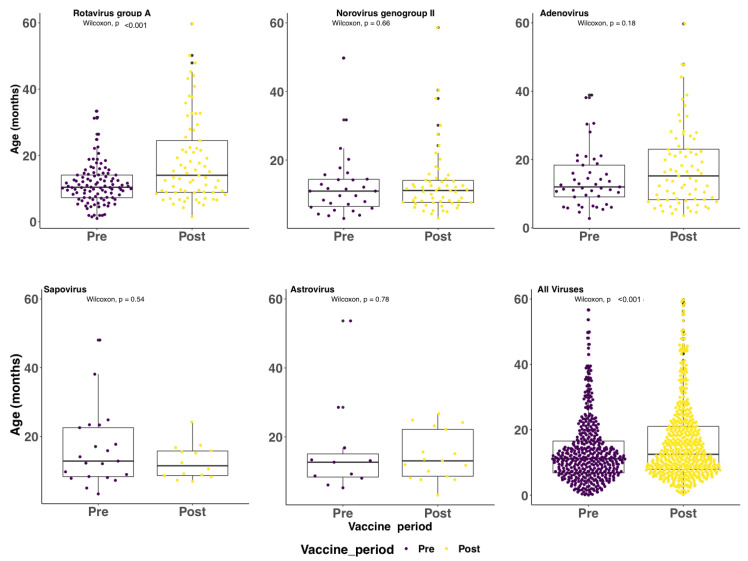
Distribution of age in months among cases under 5 years of age admitted to KCH Kenya with diarrhea symptoms pre- and post-vaccine introduction.

Notably, RVA and sapovirus cases in the post-vaccine introduction period had statistically significant lower and higher median Ct values, respectively, compared to the pre-vaccine period (Wilcoxon, *p* value < 0.001); see Figure 3. This was not observed for the other three screened viruses pre- and post-rotavirus vaccine introduction. The median age of the RVA positive cases was significantly higher for the post-vaccine introduction period (14.0 months) compared to the pre-vaccine introduction period (10.4 months) (Wilcoxon, *p* < 0.001). A similar shift was not observed for the other viruses; see Figure 4.

### 2.4. Virus Coinfections (i.e., Two or More Viruses in a Single Specimen)

These were detected in 47 specimens (4.8%). In 583 specimens (59.2%), none of the targeted viruses was detected. The prevalence of coinfections pre-vaccine was 4.4% (95% CI: 2.7–6.7%), while in the post-vaccine introduction period, this value was 5.7% (95% CI: 3.9–8.0%), *p* = 0.454. RVA and astrovirus were the most common coinfections in the pre-vaccine introduction period (*n* = 6), while in the post-vaccine introduction period, it was RVA and adenovirus (*n* = 15); see Table 4.

### 2.5. Circulating RVA Genotypes Pre- and Post-Vaccine Introduction

G1P[8] was the predominant RVA genotype pre vaccine introduction. However, in the post-vaccine introduction period, the predominant genotypes were G2P[4] (2016) and G3P[8] (2019); see Table 5.

### 2.6. Seasonality of the Detected Viruses

**Figure 5:**
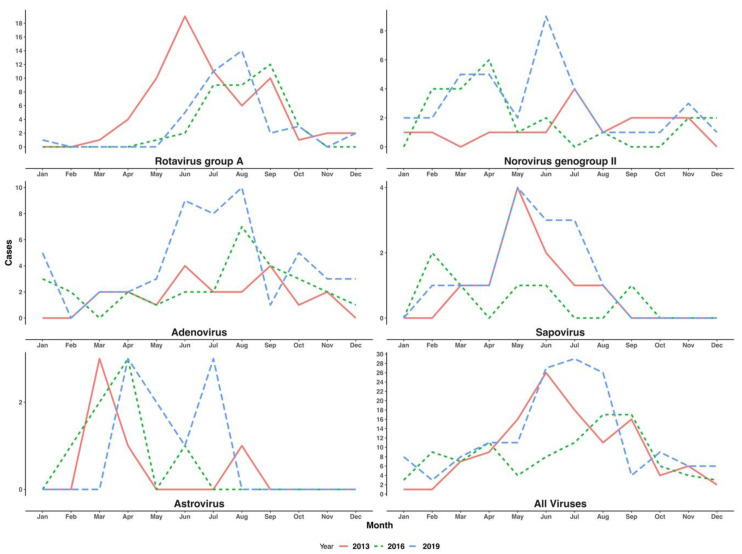
The frequency of detection of RVA, adenovirus, norovirus GII, astrovirus and sapovirus by month in children under 5 years of age admitted to KCH Kenya with diarrhea in 2013, 2016 and 2019.

We constrained this analysis to the years 2013, 2016 and 2019, where >70% of the eligible patients had been analyzed. Pre-vaccine introduction (in 2013), for RVA, there were two peak months, in June and September. However, post-vaccine introduction (in 2016 and 2019), there was only a single peak month for RVA in September and August, respectively. For norovirus GII, cases were observed throughout the year, with peak months varying from year-to-year, in July, April and June in 2013, 2016 and 2019, respectively. Similarly, adenovirus cases appeared to occur throughout the year, with two peak months in 2013 (June and September) and one peak month in 2016 and 2019 (August for both). For sapovirus and astrovirus, we observed less than five cases monthly between January and August and no cases in the last quarter of each the three years; see Figure 5.

### 2.7. Primer/Probe Mismatches with Contemporary Sequences

**Figure 6:**
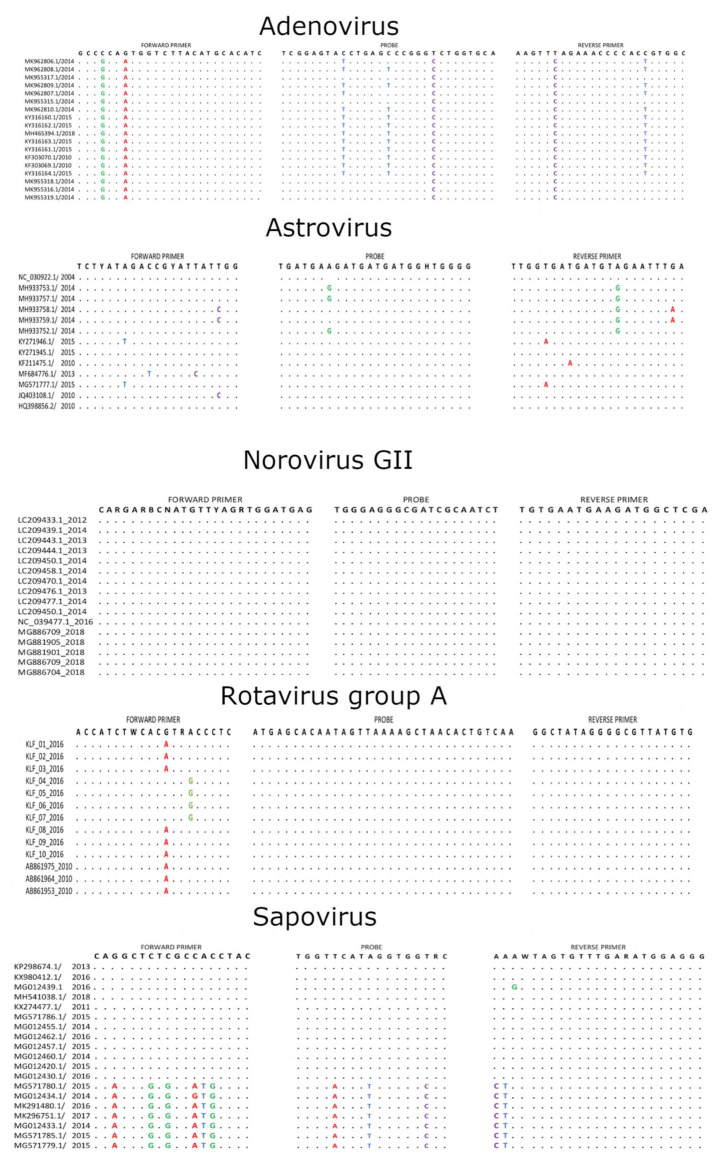
The primers and probes target sites for RVA, adenovirus and norovirus GII, sapovirus and astrovirus were aligned using MAFFT v.7.31313 and the alignments were trimmed to the region of the primer and probe target sites. Nucleotide differences between the expected primer and probe target sites and the viral sequences were identified and highlighted. Dots indicate identity with primer or probe sequences.

Nucleotide mismatches were observed in either or both the primers and probes and the viral target sequences for all the viruses except for norovirus GII; see Figure 6. The RVA forward primer had a G-A and A-G mismatches at positions 12 and 15, respectively. Adenovirus had two mismatches in the forward primer (C-G and G-A), three mismatches in the probe (C-T, C-T and T-C) and two mismatches in the reverse primer (T-C and C-T), and none of them were within five bases of the 3′ end. Mismatches within the sapovirus primer/probe binding sites were pronounced in sapovirus genogroup V and included six mismatches in the forward primer, three mismatches in the probe and two mismatches in the reverse primer. Some of the mismatches were within five bases of the 3′ end (forward primer: C-G, probe: T-C, reverse primer: A-C and T-C). Astrovirus primers and probe did not have pronounced mismatches present in all the sequences—rather, they had mismatches in individual sequences; see Figure 6.

## 3. Discussion

We observed a significant decrease in the prevalence of RVA in the post-vaccine introduction period in KCH, concurring with findings of a recent multi-site study in Kenya that reported RVA vaccine effectiveness of ~64% (95% CI: 35–80%) and a reduction in rotavirus-associated hospital admissions two years post-vaccine introduction of ~80% (95% CI: 46–93%) [9,11]. Note that Kenya rotavirus vaccine coverage was considered medium in 2018 (70–79%) [12]. Our pre- and post-vaccine introduction analysis observed a significant increase in the prevalence of norovirus GII in KCH post-rotavirus vaccine introduction, as similarly observed in the United States, Nicaragua and Bolivia following RVA vaccine introduction [13,14,15]. It is unclear if this has been driven by an established biological interaction between these two viruses or that this reflects natural norovirus GII fluctuation in prevalence across multiple years.

The shift in the predominant genotypes pre- and post-vaccine introduction from G1P[8] to G2P[4] in 2016 and G3P[8] in 2019 in our setting has also been described elsewhere, e.g., in Belgium, Madagascar and Ethiopia [16,17,18]. G3P[8] was the predominant genotype in this setting in 2018 [19] and it continued being the dominant genotype in 2019. Although these dominant post-vaccine genotypes are either partially or fully heterotypic to the Rotarix G1P[8] strain, in their surface exposed immunodominant proteins, there is not enough evidence yet to directly attribute their increased incidence to vaccine introduction [20]. Additional analysis will help to bring better understanding on the reason behind their dominance.

Despite RV vaccine introduction in Kilifi, Kenya, no significant difference was observed in the discharge outcome for all causes of diarrhea pre- and post-rotavirus vaccine introduction. We suggest two explanations for this. Firstly, the majority of the children who were eligible to be in this study and died did not have a sample collected to determine their RVA and other enteric pathogens’ status. Secondly, inpatient mortality of children treated for diarrhea in Kilifi County Hospital has been previously found to be predicted by a positive HIV test, bacteremia and poor nutritional status [21]. This may have not changed pre- or post-introduction of rotavirus vaccination.

RVA Ct values were decreased in post-vaccine samples compared to pre-vaccination years. This was despite RVA disease severity remaining unchanged between the two periods. Different extraction methods were used to process the samples between 2003, 2013 and 2016, 2019. However, according to Liu et al., the difference in the extraction methods for enteric pathogen studies is not significant, except for norovirus GII, which showed a higher Ct value with kits targeting RNA purification alone compared to those targeting total nucleic acid (TNA) (difference within 1 Ct value). Different extraction kits were used in this study because raw stool samples from 2003 to 2016 were already destroyed following a directive by the WHO in 2016 that was part of the larger global polio eradication effort.

It has been previously noted the introduction to rotavirus vaccines may result in the shift of diarrhea disease burden to slightly older age groups [20]. Our study found a significant increase in the median age of diarrhea cases post-vaccine introduction (12.5 months) compared 11.2 months pre-introduction. This in part may be explained by the higher immunity at both individual and population levels against rotavirus that wanes as children grow older.

On local seasonality patterns, in each year, a peak month(s) of occurrence was observed for RVA, norovirus GII, sapovirus and adenovirus but not astrovirus. The Kilifi area has a tropical climate with two rainy seasons; the main rains usually peak in May (up to July) while the short rains usually peak in November (can run from October to December). RVA and adenovirus appeared to peak in the dry months while norovirus GII and sapovirus peaked in the rainy season. Similar patterns in the seasonality of RVA, adenovirus, norovirus GII and sapovirus have been observed elsewhere [22,23,24,25]. The seasonality of astrovirus is not well described.

The performance of qPCR assays can be impacted by mismatches within the last five bases at the 3′ end of primers and probe or/and the number of mismatches being more than five in the primers and probe [26,27]. The mismatches observed in the primer and probe binding sites of adenovirus, astrovirus and sapovirus may have impaired the real-time PCR function by blocking the amplification or increasing the quantification cycles. Consequently, this may have impacted the estimated frequency of detection of these viruses. Unlike for RVA, the magnitude of the mismatches in qPCR function could have been shown better using recent local sequences of the other viruses.

This study had limitations: firstly, we did not analyze healthy children in the community to inform on the background prevalence of the five viruses in our study population. Secondly, the adenovirus assay was not specific to type 40/41 alone; thus, some of the adenoviruses detected may not be associated with diarrhea. Thirdly, a significant number of eligible cases were not sampled, including those who died before sampling. This potentially biased prevalence of the screened pathogens in the study population. Fourthly, extracting TNA from samples after many years of storage could lead to lower Ct values due to deterioration. Finally, the seasonality of examined pathogens will be best described if we examine more years.

In conclusion, we found a significant decline in the prevalence of rotavirus in hospitalized children in coastal Kenya after rotavirus vaccine introduction. This finding reinforces evidence of the continued benefit of rotavirus vaccination in this setting. Concomitantly, there has been a surge in norovirus GII prevalence, but the factors driving this increase are unclear and will require future investigation. The observation that the screened viruses peak at different times of the year also would benefit further investigation in order to understand drivers of their transmission and inform the design of effective intervention measures.

## 4. Materials and Methods

### 4.1. Study Site and Population

This study was undertaken at KCH, a referral hospital serving the Kilifi County population, which is majorly a rural population. We utilized stool specimens collected during routine surveillance of rotavirus in children with diarrhea as one of their illness symptoms, aged below five years and admitted to KCH [9,11]. Diarrhea was defined as observation of three or more loose stools in the preceding 24-h period. In this study, we selected two pre-vaccine years (2003 and 2013) and two post-vaccine years (2016 and 2019) for analysis. A stool specimen was collected from children who met the diarrhea case-definition following parental or guardian consent. The study protocol was approved by the Scientific and Ethics Review Unit (SSC#2861 and SERU#CGMRC/113/3624) based at KEMRI, Nairobi, Kenya.

### 4.2. Laboratory Methods

Irrespective of their previously determined rotavirus status, TNA were extracted from 0.2 g of 2003 and 2013 specimens (or 200 µL if liquid) using the cador Pathogen 96 QIAcube HT Kit (Qiagen, Manchester, UK). For 2016 and 2019 specimens, TNA were extracted using QIAamp Fast DNA Stool Mini kit (Qiagen, Manchester, UK) as per the manufacturer’s instructions. Fecal specimens from the post-vaccine period (0.2 mg or 200 µL) were subjected to bead beating prior to TNA extraction and collected in a 200 µL of elution buffer [28].

The TNA extracts were screened for the five viruses by a two-step real-time RT-PCR assay [29]. First, cDNA was synthesized in a total volume of 20 µL using random hexamers and 5µL of TNA using the Omniscript Reverse Transcriptase kit (Qiagen, Manchester, UK), as per the manufacturer’s instructions. Two µL of the cDNA was henceforth used for real-time RT-PCR in a total volume of 20 µL using the QuantiFast RT-PCR Kit (Qiagen, Manchester, UK) and run on the ABI 7500 Real-Time PCR System (Applied Biosystems, Foster City, CA, USA). Primers and probes were adopted from previously published work [30]. The presence of nucleotide mismatches in the primer and probe binding sites was investigated by aligning the primers/probes to genomic sequences deposited in GenBank from 2010 to 2019, using MAFFT v.7.313 [31]. The adenovirus probe/primer pair used in this study detected adenovirus serotypes beyond type 40/41. We used three Ct cut-off values (<40.0, <35.0 and <30.0) to define positive samples. Samples that were positive for RVA in 2003, 2013, 2016 and 2019 were processed for RVA genotyping using VP4 and VP7 RT-PCR, followed by either dideoxy sanger sequencing, as described elsewhere [19], or next-generation sequencing on the Illumina Miseq platform [32].

### 4.3. Statistical Analysis

All statistical analyses were performed using R version 3.6.1 [33]. Prevalence was defined as the proportion of these viruses in a hospital-admitted diarrhea patient population during the study period in Kilifi, Kenya. Means and medians of continuous variables were compared using a Kruskal Wallis and Wilcoxon rank-sum test, respectively. Binary data were summarized using proportions and comparisons between groups made using χ^2^ statistics. A *p* value of <0.05 was considered statistically significant. Diarrhea severity in RVA positive cases pre- (year 2013) and post- (years 2016 and 2019) was assessed using the Vesikari Clinical Severity Scoring System Manual [10], with a modification in the treatment parameter. If the participant was given oral rehydration therapy or intravenous fluid therapy, they received a score of one or two, respectively.

## Figures and Tables

**Table 1:** Characteristics of children under 5 years of age admitted to Kilifi County Hospital (KCH), coastal Kenya, with diarrhea symptoms that were sampled versus those who were not sampled in the study.

Characteristics	All Subjects	Sampled (%)	Not Sampled (%)	*p*-Value
**Total Admissions**	2156	1397 (64.8)	759 (35.2)	
**Admissions Per Year**				
**2003**	1007 (46.7)	587 (42.0)	420 (55.3)	
**2013**	332 (15.4)	254 (18.2)	78 (10.3)	
**2016**	334 (15.5)	257 (18.4)	77 (10.1)	
**2019**	483 (22.4)	299 (21.4)	184 (24.2)	
**Gender**				0.838
**Male**	1262 (58.5)	815 (58.3)	447 (58.9)	
**Female**	894 (41.5)	582 (41.7)	312 (41.1)	
**Age**				
**Median (IQR)**	12.4 (7.7–20.5)	11.7 (7.4–19.7)	13.8 (8.5–22.1)	**<0.001**
**Mean (SD)**	15.7 (11.4)	15.0 (11.1)	16.9 (12.0)	**<0.001**
**Age Group**				**0.001**
**0** **–** **11 Months**	1045 (48.4)	718 (51.4)	326 (43.0)	
**12** **–** **23 Months**	716 (33.2)	444 (31.8)	272 (35.8)	
**24** **–** **59 Months**	396 (18.4)	235 (16.8)	161 (21.2)	
**Discharge Outcome (*n* = 2153) ^#^**				**<0.001**
**Alive**	1918 (88.9)	1306 (93.5)	612 (80.5)	
**Dead**	235 (10.9)	89 (6.4)	146 (19.3)	

SD means standard deviation; IQR means interquartile range. Not sampled: sample was not collected due to lack of consent, time-up, death and others. ^#^ Discharge outcome data for three subjects were missing.

**Table 2:** Characteristics of children under 5 years of age admitted to KCH, coastal Kenya, with diarrhea symptoms and tested pre-vaccine introduction versus those tested post-vaccine introduction.

Characteristics	Total	Pre-Vaccine Introduction (%)	Post-Vaccine Introduction (%)	*p*-Value
**Number of Samples Tested**	984	454 (46.1)	530 (53.9)	
**Samples Tested (Year)**				
**2003**	223	223	-	
**2013**	231	231	-	
**2016**	239	-	239	
**2019**	291	-	291	
**Gender**				0.847
**Male**	570 (57.9)	261 (57.5)	309 (58.3)	
**Female**	414 (42.1)	193 (42.5)	221 (41.7)	
**Age**				
**Mean (SD)**	15 (11.2)	13.4 (9.9)	16.3 (12)	<0.001
**Median (IQR)**	11.7 (7.3–19.3)	11.2 (6.8–16.5)	12.5 (7.9–21)	<0.001
**Age group**				0.003
**0–11 Months**	505 (51.3)	252 (55.5)	253 (47.7)	
**12–23 Months**	323 (32.8)	148 (32.6)	175 (33.0)	
**24–59 Months**	156 (15.9)	54 (11.9)	102 (19.3)	
**Disease Severity in RVA Cases = *n* (139)**				
**Mild**	12 (8.6)	7 (10.6)	5 (6.8)	0.441
**Moderate**	50 (36.0)	26 (39.4)	24 (32.9)	
**Severe**	77 (55.4)	33 (50)	44 (60.3)	
**Discharge Outcome = *n* (982) ^#^**				0.556
**Alive**	925 (94.2)	425 (93.6)	500 (94.7)	
**Dead**	57 (5.8)	29 (6.4)	28 (5.3)	

SD means standard deviation; IQR means interquartile range; RVA means rotavirus group A. Values given are the counts and percentages are provided in brackets. ^#^ Discharge outcome for two subjects was missing. Disease Severity Was Calculated Using the Vesikari Clinical Severity Scoring System Manual [10].

**Table 3:** Comparison of the prevalence of viral detection in children under 5 years of age admitted to KCH Kenya with diarrhea symptoms pre- and post-rotavirus vaccine introduction.

Viruses Detected	Total	Pre-Vaccine Introduction (%)	Post-Vaccine Introduction (%)	*p*-Value
**Samples Tested**	984	454 (46.1)	530 (53.9)	
**Rotavirus Group A**	179 (18.2)	106 (23.3)	73 (13.8)	<0.001
**Adenovirus**	120 (12.2)	45 (9.9)	75 (14.2)	0.053
**Norovirus GII**	88 (8.9)	30 (6.6)	58 (10.9)	0.023
**Astrovirus**	28 (2.8)	11 (2.4)	17 (3.2)	0.585
**Sapovirus**	35 (3.6)	21 (4.6)	14 (2.6)	0.133

**Table 4:** Coinfections pre- and post-rotavirus vaccine introduction. RVA stands for rotavirus group A, ADV stands for adenovirus, NOR GII stands for norovirus GII, ASV stands for astrovirus and SAP stands for sapovirus.

PATHOGEN COINFECTION	PRE-VACCINE INTRODUCTION	POST-VACCINE INTRODUCTION
**RVA & NOR GII**	1	2
**RVA & ADV**	2	15
**RVA & ASV**	3	0
**RVA & SAP**	6	1
**NOR GII& ADV**	3	4
**NOR GII & ASV**	0	1
**NOVGII & SAP**	2	1
**ADV & ASV**	1	3
**ADV & SAP**	1	1
**ASV & SAP**	1	2

Abbreviations used for viruses as in Figure 3.

**Table 5:** Frequency of RVA genotypes detected in coastal Kenya pre- (2003 and 2013) and post- (2016 and 2019) vaccine introduction.

Year	2003		2013		2016		2019	
	No. of Cases	%	No. of Cases	%	No. of Cases	%	No. of Cases	%
**RVA Positive**	40		66		36		37	
**Genotyped**	2	5.0	48	72.7	34	94.4	36	97.3
**Genotypes**								
**G1P[8]**	1	50.0	43	89.6	5	14.7	1	2.8
**G2P[4]**	-	-	2	4.2	29	85.3	-	-
**G3P[8]**	-	-	1	2.1	-	-	34	94.4
**G9P[8]**	1	50.0	1	2.1	-	-	-	-
**G10P[8]**	-	-	1	2.1	-	-	-	-
**G8P[8]**	-	-	-	-	-	-	1	2.8
